# Computational modeling of human multisensory spatial representation by a neural architecture

**DOI:** 10.1371/journal.pone.0280987

**Published:** 2023-03-08

**Authors:** Nicola Domenici, Valentina Sanguineti, Pietro Morerio, Claudio Campus, Alessio Del Bue, Monica Gori, Vittorio Murino

**Affiliations:** 1 Uvip, Unit for Visually Impaired People, Istituto Italiano di Tecnologia, Genoa, Italy; 2 University of Genova, Genoa, Italy; 3 Pavis, Pattern Analysis & Computer Vision, Istituto Italiano di Tecnologia, Genoa, Italy; 4 Visual Geometry and Modelling, Istituto Italiano di Tecnologia, Genoa, Italy; 5 University of Verona, Verona, Italy; 6 Huawei Technologies Ltd., Ireland Research Center, Dublin, Ireland; University of Minnesota, UNITED STATES

## Abstract

Our brain constantly combines sensory information in unitary percept to build coherent representations of the environment. Even though this process could appear smooth, integrating sensory inputs from various sensory modalities must overcome several computational issues, such as recoding and statistical inferences problems. Following these assumptions, we developed a neural architecture replicating humans’ ability to use audiovisual spatial representations. We considered the well-known ventriloquist illusion as a benchmark to evaluate its phenomenological plausibility. Our model closely replicated human perceptual behavior, proving a truthful approximation of the brain’s ability to develop audiovisual spatial representations. Considering its ability to model audiovisual performance in a spatial localization task, we release our model in conjunction with the dataset we recorded for its validation. We believe it will be a powerful tool to model and better understand multisensory integration processes in experimental and rehabilitation environments.

## Introduction

In everyday life, we are constantly stormed by various sensory stimulations. At any given moment, we perceive the environment through most of, if not all, our senses, and our brain must segregate or integrate sensory information to build a functional representation of the surroundings. Imagine you are attending a concert, standing still in the cheering crowd. At some point, a groovy guitar solo starts, and you turn your gaze from the unrestrained singer to the musicians: unluckily, two guitarists are simultaneously playing, and you want to understand which one is playing the solo. At first glance, interpreting a similar situation might seem natural; from a computational standpoint, however, our brain must face at least two issues to exploit the stream of incoming sensory inputs and correctly identify the playing guitarist.

On the one hand, the brain must solve the recoding problem: since different sensory signals are initially represented through different frames of reference, it has been suggested that they must be set to a common metric before being integrated [1–7]. On the other hand, the brain must infer statistical probabilities for each sensory information combined into a multisensory representation [8–10], depending on the context and the information itself—an issue known as the statistical inference problem. Visual information is often more informative than auditory when localizing the position of an object [11], and because of this, it often ‘attracts’ the latter. This specific interaction can be easily understood if we think of a ventriloquism show. The voice comes out from the showman’s mouth during the performance, but our eyes see the puppet’s lips moving, and we therefore perceive the sound as it is coming from the puppet’s mouth. Indeed, since visual information is more reliable than auditory information in this scenario, our brain combines the available sensory cues and perceives the voice as it came out from the marionette. Not surprisingly, this effect is known in neuroscience as the ventriloquist effect [12].

Considering the multisensory problems from a modeling perspective, it has been proposed that humans resort to Bayesian strategies to maximize optimal integration, as extensive behavioral evidence advocates for their ability to make Bayesian inferences when integrating multisensory inputs [8, 11, 13, 14]. This inevitably leads to two dutiful considerations: first, our perceptual behavior is tightly linked to our ability to infer Bayesian probabilities, but inferences following Bayes’ theorem are linearly determined (i.e., the perceptual output is the weighted sum of the single sensory inputs), while information reliability determines each weight, with reliability being the inverse of sensory variance [15, 16]. Second, the statistical inference problem can be explained considering Bayes-optimal integration, but the recoding problem cannot. Specifically, no study included the recoding problem within a Bayesian framework, and a purely linear approach does not account for all the above multisensory integration issues [1].

In recent years, computational approaches have been developed to condense and solve most multisensory integration problems [1, 17–19]. For example, Pouget and colleagues developed a three-layers recursive neural network based on both basis function and attractor dynamics, tackling the statistical inference and the recoding problem. The authors tried to explain specific neural mechanisms with such a network, mainly referring to gain and partially shifting receptive fields [1]. Despite its undeniable elegance, the model assumes that all sensory information comes from the same physical object. Therefore, it is hard to generalize its structure to any other situation where more than one signal’s source is available. In recent work, Song and colleagues developed a recurring neural network (RNN) to investigate the computational mechanisms behind large neural populations, including multisensory integration [20]. The authors improved their RNN following refined Dale’s principle [21], for which cortical neurons exhibit pure inhibitory or excitatory post-synaptic influence at a time, thereby significantly increasing its biological plausibility. However, none of the tasks used to validate the RNN were tailored to integrate displaced audiovisual information for source localization, as none of them aimed at evaluating multimodal spatial localization, nor any of the stimuli was spatially informative.

Here, we propose a computational representation of the recoding and the statistical inference problems of multisensory integration in a spatial task, developing a slightly different empirical approach focusing on Bayesian inferences. We based our architecture on the human’s ability to localize the position of a stimulus with respect to their own (i.e., targeting a specific set of spatial abilities), replicating a basic design that has been extensively used to infer spatial perception across the lifespan [22]. We first modeled and trained a neural architecture to resemble humans’ ability to localize visual, auditory, and audiovisual stimuli. To test its ability to integrate auditory and visual signals, we considered the architecture’s behavior when conflicting audiovisual information was displayed, i.e., when auditory and visual stimulations were simultaneously presented but spatially displaced, such as in the ventriloquist illusion [11]. Then, we compared the architecture’s performance with the performance of the human observer to validate the former’s phenomenological plausibility. Our model allows the evaluation of single-sensory weights that can be considered to infer multisensory performance, even though it can be less informative at the neuronal level compared to the other models available in the literature. Similar to the human observer, our architecture was then evaluated considering Bayesian inferences, assuming specific probability distributions for each information in input. Differently from canonical machine learning applications and similarly to the approach fostered by Song and colleagues [20], our goal was not to maximize at all costs the model’s performance but to replicate a human-like perceptual behavior. With the current work, we propose an easy-to-use, empirically reliable neural architecture that can achieve optimal statistical integration when combining audiovisual stimuli for source localization. Its implications are further discussed, considering possible future applications and additional strengths, such as the shared dataset containing an accurate replica of the stimuli usually involved when testing human observers using psychophysics, allowing a direct comparison and an overall better understanding of perceptual behavior.

## Materials and methods

### Human observer

#### Participants

A total of nine participants (27.44 ± 2.55 y, 6F), all naïve to the purpose of the study, were recruited to compare their performance with the neural architecture’s one. All participants had normal hearing and normal or corrected-to-normal vision, while none reported a history of neurological, cognitive, or sensory disorders. Before testing, each participant signed written informed consent. Data collection was performed in Genova (Italy), at the Istituto Italiano di Tecnologia, in a dark room specifically disposed of for the occasion. Testing procedures were previously approved by the local ethical committee (Comitato Etico, ASL 3, Genova) and were developed following the declaration of Helsinki.

#### Apparatus and stimuli

**Fig 1:**
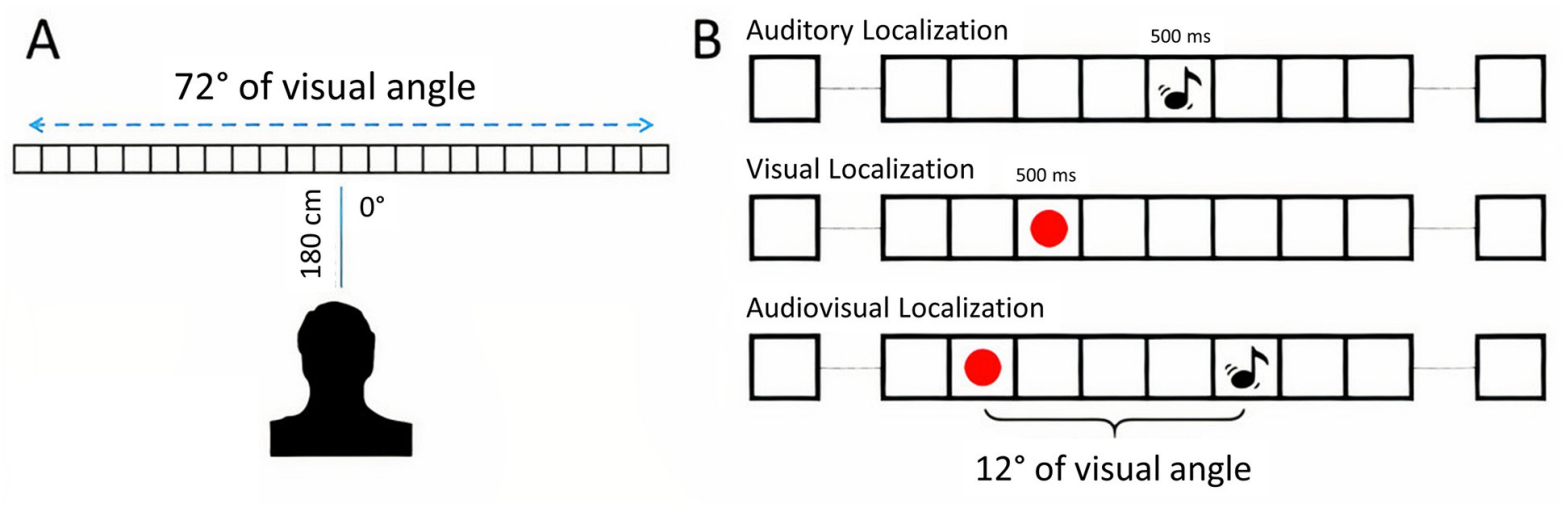
A) 24-modules array used to deliver stimuli in all three localization tasks. Participants sat at 180 cm of viewing distance, exactly in the middle of the array, with the latter spanning ± 32 degrees of visual angle. B) Experimental paradigm, describing the auditory, visual, and localization tasks. In the latter, the displacement between stimuli was always ±4 modules (±12° of visual angle at a viewing distance of 180 cm).

Participants sat in a quiet, dark room, 180 cm away from the center of a 24 modules custom-made array (Fig 1A) spanning ± 36° of visual angle (with 0° being the center of the array, negative and positive values indicating leftward and rightward position, respectively). All testing procedures took place in total darkness so that the array was not visible to the participants, excluding the possibility that responses were influenced by contextual cues (such as the array’s silhouette). Each module could deliver either visual or auditory stimulation. Visual stimuli were red flashes with a diameter of 3° of visual angle at participant’s viewing position, while auditory stimuli were 2 kHz sine wave pulses with 60 dB Sound Pressure Level (SPL). Every module could deliver only one stimulus at a time, i.e., a module could produce either a sound or a flash, never both simultaneously (even though participants were unaware of that). The array was linked to the computer used to run the experiment through a USB cable. The connection between the array and the laptop was also powered via a dedicated host. The experiment was developed and run using MATLAB (v. 2013b).

#### Psychophysical tasks

Participants completed three different behavioral tasks (Fig 1B): a visual, auditory, and audiovisual localization task. In each session, regardless of the sensory modalities involved in the task, participants had to indicate the position of a 500 ms stimulus pseudo-randomly displayed on the array. At the end of each trial, a persistent LED light was displayed on a random array module to report the stimulus’s position. Participants moved such light with a mouse until they reached the desired spatial location. Then, a simple click registered the response (initially coded considering the id of the selected module). Two seconds after the answer was given, the subsequent trial started. In the auditory task, stimuli were placed ± 4.5°, ± 13.5°, or ± 22.5° with respect to the observer’s point of view. Each stimulus’s position was repeated ten times, leading to 60 trials for the auditory localization task. In the visual task, stimuli were placed ± 1.5°, ± 7.5°, ± 10.5°, ±16.5°, ±25.5°, or ± 34.5° with respect to the observer’s point of view. Each stimulus position was repeated five times, leading to 60 trials for the visual localization task. In the audiovisual localization task, stimuli were a combination of a spatially displaced flash and sound drawn from the spatial positions used in the two single sensory tasks. Sounds were placed ± 4.5°, ± 13.5°, or ± 22.5° with respect to the observer’s point of view, similar to what was already done in the auditory condition, while flashes were always displayed ± 12° from the position of the sound, replicating spatial positions used in the visual condition. Therefore, for each sound position, two different bimodal trials were implemented: one in which the flash was placed leftward and one in which the flash was placed rightward. In the end, each possible bimodal combination (sound and Led placed either 12° leftward or rightward) was repeated five times, leading to 60 trials. Since we wanted to evaluate how vision attracted auditory perception (similar to what happens during a ventriloquism show), we asked participants to report the sounds’ apparent position in the audiovisual localization task.

Participants performed a total of 180 trials, and the whole testing procedure required around 30 minutes, depending on the participant, and was completed in a single session. The order of localization tasks was randomized across participants to avoid any possible interference due to undesired familiarization effects.

#### Audiovisual localization model

To predict audiovisual localization, we referred to one of the hierarchical Bayesian causal inference models described by Rohlf and colleagues [22]. We opted for the model including an averaging strategy (Causal Inference-Model Averaging, CI-MA) as it best predicts audiovisual spatial integration for spatial localization. As with any other Bayesian model, the CI-MA evaluated the probability distributions of both auditory and visual signals, including in the estimation any prior belief, and combined them to infer the position of the audiovisual stimulus directly. For such inference, consider S_a_ and S_v_ the actual position of the auditory and visual signals, respectively. When encoded by the brain, sensory noise inherently distorts this information. Both stimuli positions are estimated using the representation X_a_ and X_v_ of S_a_ and S_v_’s physical positions. Therefore, X_a_ and X_v_ change across observations and different trials. As a general rule, the CI-MA model infers the causal structure (C) of the cues using Bayes’ formula:

$$p\left(C|X_{a},X_{v}\right)=\frac{p\left(X_{a}X_{v}|C\right)p\left(C\right)}{p\left(X_{a},X_{v}\right)}$$ (1)

To model spatial localization in our study, we assumed a single common cause C = 1, as it denotes the integration of both sensory cues into a single percept, i.e., we assumed no cue segregation since the displacement between auditory and visual cues was not sufficient to avoid integration [22]. With C = 1, the optimal audiovisual estimate was obtained by integrating auditory and visual cues weighting them considering their reliabilities, the inverse of their variances. Therefore, the optimal estimate was obtained by:

$$S_{a}\left(C=1\right)=S_{v}\left(C=1\right)=\frac{\frac{X_{a}}{\sigma_{a}^{2}}+\frac{X_{v}}{\sigma_{v}^{2}}+\frac{\mu_{P}}{\sigma_{P}^{2}}}{\frac{1}{\sigma_{a}^{2}}+\frac{1}{\sigma_{v}^{2}}+\frac{1}{\sigma_{P}^{2}}}$$ (2)

With μ_P_ and σ_P_ being the mean and the standard deviation of the prior probability, and σ_a_ and σ_v_ being the standard deviation of the Gaussian noise shaping single auditory and visual signals centered around X_a_ and X_v_, respectively. To obtain values for the single-sensory estimations, we individually fitted a Gaussian function into humans’ averaged data for auditory and visual localization conditions (Fig 4A and 4B). We used the parameters obtained by fitting Gaussian curves into averaged human observers’ data (see Results section).

We assumed no prior spatial bias during audiovisual integration (hence, we fixed μ_P_ = 0), and σ_P_ was the only free parameter to be varied. Then, following the model designed by Rohlf and colleagues, we drew 10,000 Monte Carlo samples for each possible {X_a_, X_v_} centered at S_a_ and S_v_ and with standard deviations σ_a_ and σ_v_, respectively. We repeated the procedure considering the conditions where the visual stimulus was to the left or the right of the auditory stimulus in separate sessions. Then, we binned response distributions with a 3° bin-width range to create a correspondence with data obtained with the neural architecture and human observers, and we evaluated the total proportion of relative responses.

Once we modeled optimal performance, we compared it with both empirical data sets. To assess how well the model predicted the observed pattern of responses, we evaluated the Normalized Root Mean Square Error (NRMSE) according to the following formula:

$$NRMSE=\frac{\sqrt{\frac{\sum_{i=1}^{n}\left(\hat{y_{i}}-y_{i}^{2}\right)}{n}}}{\bar{y}}$$ (3)

Where *n* is the number of trials, ${\hat{y}}_{i}$ and *y*_*i*_ are the estimated and the actual position of the stimulus for the i-th trial, and $\bar{y}$ is the range of the measured data (obtained by calculating the difference between y_max_ and y_min_). Generally, lower NRMSE values indicate less residual variance and a better model prediction. Suppose the neural architecture can integrate audiovisual information in a human-like fashion. We expect a similar NRSME when comparing its performance and the human observers with the resulting mathematical simulation.

### Neural architecture

#### Audio, visual, and audiovisual models

For our audio, video, and audiovisual models, we employ three versions of U-Net architecture: an autoencoder model with a skip connection [23]. We employed this popular generative model to design a method for localizing audiovisual stimuli, drawing inspiration from human pose estimation methods [24], which use architectures similar to U-Net to obtain heat maps localizing human joints. Additionally, U-Net architecture is suitable for our task, as it allows us to learn the correspondence between input data and output localization maps in the pixel space. In fact, it has been proven in the computer vision literature that for localizing points in the space, modeling spatial uncertainty through a heatmap works better than simply regressing a 2d position in the space or a 1d position in an array [25].

**Fig 2:**
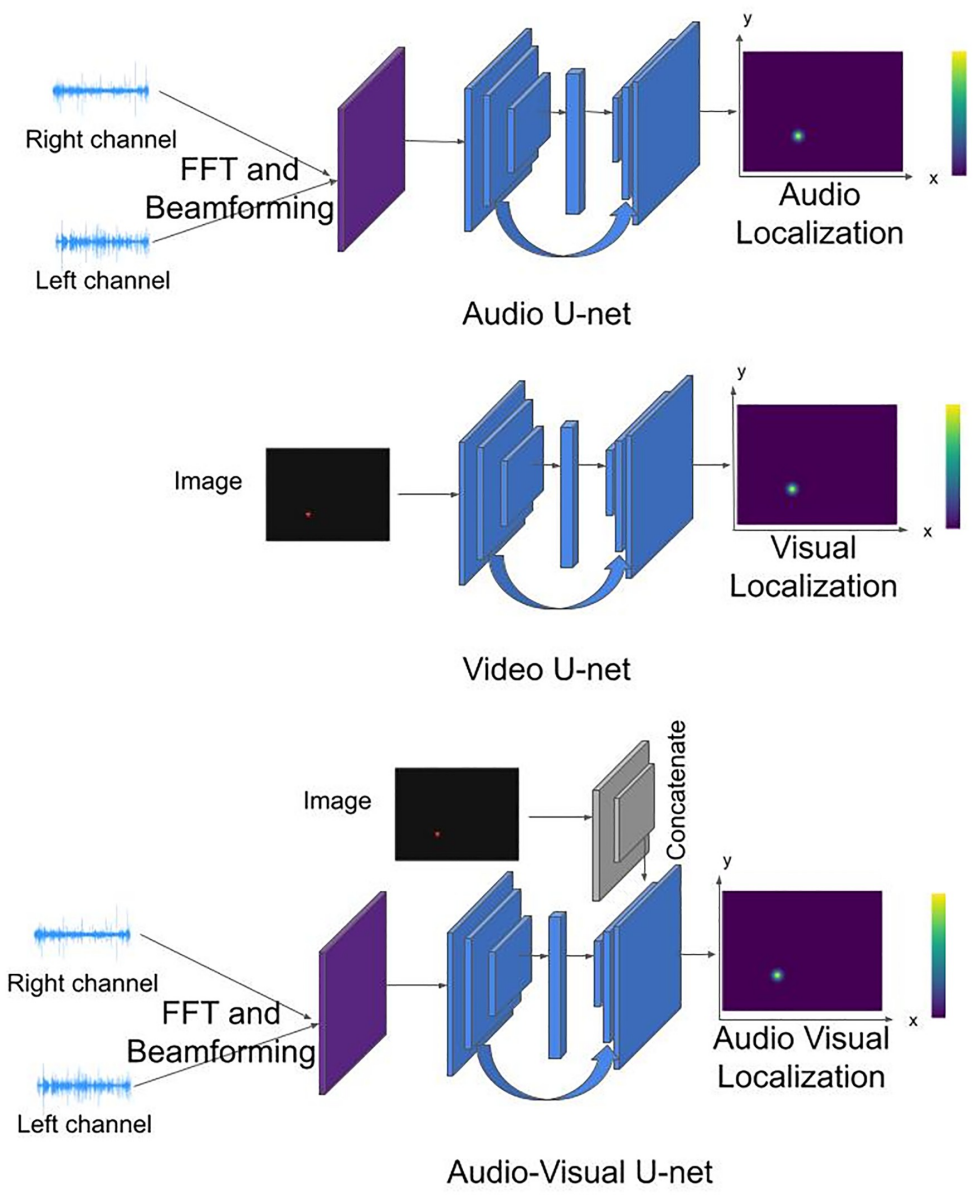
The considered models: Audio, video, and audio-visual U-net with the corresponding inputs: Audio features obtained from beamforming preprocessing, image of the LED in the dark room, or both. The networks are able to generate a 2D localization map of the visual stimulus in the case of Video U-net, or an audio impulse in the case of Audio and Audio-Visual U-net.

The results of the video U-Net are more reliable than those of the audio U-Net since images have spatial cues which are easier to extract. Concatenating visual features to audio U-Net, we obtain the audiovisual U-Net, shown in Fig 2. All the models generated as output the spatial localization map of the stimulus, either the audio signal (for audio and audiovisual model) or the light, with a fixed 224x298 pixel map from which we can find the predicted module of the array.

Being a neural architecture, U-Net is data-driven and has to be trained with data, as humans learn from experience. Therefore, we recorded videos in the same experimental conditions as those performed on humans to pass them to our model. Before being given as input to the architecture, signals were preprocessed (see Dataset section). In the preprocessing, we extracted the Mel Frequency Cepstral Coefficients (MFCC) from the waveforms of two microphones via beamforming algorithm (for the audio signals), and we removed the residual light coming from behind the curtains to avoid possible confusion from the visual network (for the visual signals). The preprocessed inputs were then given as input to the neural architecture, whose output is a 2D Gaussian localization map, which ideally should have its maximum value on the stimulus (either audio or LED) coordinate. In the next section, we explain the dataset collection and the training of our models in more detail.

#### Dataset

We collected a dataset designed to replicate the experimental procedure implemented with humans. To do so, we reused the experimental setup developed to test human observers, thus involving the same array of speakers and delivering an identical set of auditory, visual, and audiovisual stimulations. Then, we positioned our optical acoustic device at a 180 cm distance to the center of the array, mirroring the position at which human observers performed the localization tasks. The optical acoustic device is composed of a planar array of 0.45 × 0.45 meters, including 128 MEMS low-cost digital microphones (displaced according to an optimized aperiodic layout) and a video camera, which has no distortion at the edges, at the center of the planar array. Using such a device, we collected both the images and two audio microphone waveforms to resemble the data acquired by human vision and hearing. From the 128 microphones included in the array, we chose two of them placed horizontally at a distance of 40 cm from each other, to receive the two audio signals with enough delay. Then, we apply beamforming algorithm to estimate the sound source’s direction. We finally compress the spectral information via Mel-Frequency Cepstral Coefficients (MFCC), which resemble how humans perceive sound. Beamforming is a well-known signal processing technique that, given a known geometric configuration of multiple microphones, combines audio signals according to the delays with which they are received by the different microphones. Then, signals can be summed coherently, to finally estimate the spatial position from where the audio signal is coming from [25]. Even though the distance between each ear in humans is 25 cm on average, such distance was insufficient to recover a reliable localization of sound’s energy. Nevertheless, we also release data collected from closer microphones for further studies.

Recording procedures were performed in a dark room to replicate the conditions in which human observers completed the tasks. The room size was approximately 4 x 8 meters long, with no furniture but a table (with a height of 100 cm) on which the array was placed. The array was positioned at the table’s edge, closer to the observer, and was about 2 meters from the behind wall. With this setup, we feed our models with the same stimuli used in the original audiovisual localization experiments. For visual stimulation, we provided the model a 224x298x3 image replicating the LED stimuli (resizing it from the original 360x480 resolution to be suitable as network’s input, with the third dimension indicating the 3 RGB channels); as auditory stimuli, we fed a 500 ms audio trace collected with a sampling frequency of 12288 Hz by the two pre-selected microphones. We collected disjoint data for training and testing, choosing different room conditions. Using a 24-modules array, we have 576 potential visual and auditory stimuli combinations for audio and visual model training. However, to boost the synchronization for our audiovisual model, we opted to train it using only audiovisual combinations with 0, ±1, ±2 modules shift (corresponding to ±0°, ±3°, ±6° of visual angle at a viewing distance of 180 cm). We selected these parameters as an auditory spatial threshold in humans is set around 5° [26]. We proceeded this way to maintain a perceptually plausible spatial correspondence between auditory and visual stimulations (as the latter fell within the auditory threshold), thus excluding the possibility that the architecture was directly trained with audiovisual combinations that could elicit the ventriloquist illusion.

A trial is a sample of either an audio trace 500 ms long, an image, or both synchronized. In the end, including all considered combinations, we used 888 audiovisual and 4184 trials during the audio and video models training phase. For the validation, 195 trials were used, while 160 were implemented in testing procedures. More specifically, for testing, we recorded the data in two-room conditions different from the training one: the room was the same, but the position in the space of microphones and array of modules was different, keeping 180 cm distance between them. In this way, the testing was performed in a different configuration from the training, i.e., audio reverberation on the walls and illumination were different to evaluate if our model generalizes to new visual and/or auditory scenarios. The testing included 12 combinations with a shift of 4 modules between auditory and visual stimuli (± 12° of visual angle), replicating similar designs used in a previous [22] and our experiment with human observers.

Nonetheless, we need a validation set to choose how long to train our models to stop the training when the model is overfitting on training data, avoiding lower generalization and worse performance. The validation set is also used to choose hyperparameters that cannot be optimized but must be fixed manually. To prepare a validation set similar to the test set, we extracted from all 576 combinations those where there is a shift of 4 modules between light and the audio signal. The model never sees test set combinations in its training phase. We manually annotated the ground truth to train and test our models for localization, drawing a rectangular mask on the correct module position. In the processing, we compute the center of such annotation to get the center ground truth coordinates of the module and draw a 2D Gaussian centered in such position, with a maximum diameter dimension between the height and width of the annotated rectangle. This 2D Gaussian is then the ground truth visual localization map.

#### Inputs

Regarding inputs of our models, we consider a single image containing the LED light glowing in the dark or two corresponding synchronized microphone audio signals 500 ms long. We collected random visual noise during stimuli acquisition due to some light visible from the small opening at the end of curtains that confused the model in the light localization task. Refinement of the visual image consisted in removing any light above the position of the array, masking the regions with some bright color, and setting those pixels with the mean black color of the darkroom. We feed the model with the refined image.

We preprocessed right and left microphone channels to get horizontal beamforming features in the frequency (Fast Fourier Transform, FFT) space, which give audio information for different horizontal directions, and are then replicated vertically. The array of 24 modules has a fixed vertical position, which does not change; therefore, we do not need any vertical information from the audio. With our horizontal linear array, we can localize on the horizontal plane only, which is a sufficient condition for our problem, but we replicate over the vertical axis the audio coefficients to get a 2D localization map.

The audiovisual model receives this 2D audio information. Additionally, we pass to the last decoder’s layer in our model the image features obtained from the video encoder, ResNet50 [27], pre-trained on a large image dataset (ImageNet, [28]). We lastly fine-tuned it using our dataset.

#### Training

We model 2D coordinates as a set of heat maps obtained by placing a 2D Gaussian probability distribution centered at the stimulus position, a common strategy when regressing points in space [24]. It is challenging to directly regress center coordinates from the input, as we verified by performing some experiments. It becomes much easier to get a localization map and then use a softmax function over the predicted two-dimensional Gaussian distribution because we estimate a probability instead of performing regression: the loss function is more convex and easier to optimize. The state-of-the-art guidelines we follow [24] suggest generating ground-truth heat maps by applying a Gaussian kernel to each center coordinate to exploit in addition to the original ground-truth coordinates. Hence, to train our models, we employ two combined methods: the first is to estimate the visual Gaussian map, and the second is to estimate the coordinates.

The first one obtains the visual localization map employing both Kullbak-Leibler (KL) loss and mean squared error (MSE) loss between the ground-truth Gaussian centered in the correct coordinate and the 2D Gaussian distribution predicted by our network. The KL loss estimates the distance between two probability distributions, so we compute the predicted and ground-truth Gaussian softmax to get their corresponding probability density functions. We weigh the KL more than the MSE loss since this impacts the model’s ability to localize the stimuli. Additionally, while considering just MSE loss or KL loss separately, we realized that KL was working better. KL and Jensen-Shannon (JS) divergence are functional losses when we estimate probability distributions. We notice that even though many works propose to use the Jensen-Shannon divergence [29], KL performed better in our case.

The second method learns the correct center coordinate by MSE loss between correct and predicted coordinates. The predicted centers are obtained from the predicted Gaussian through soft-argmax. It has emerged as a differentiable alternative to argmax for heatmap-based human pose estimation models [30, 31]. Since it is possible to backpropagate through the soft-argmax operation, a loss is applied directly to the predicted coordinates. Thus, we compute the softmax of the 2D Gaussian distribution, multiplied by weight to help the training, enhancing the difference between the peak and the rest of the map, and we can estimate which is the most probable center from the visual map, according to the resulting probabilities.

The two training strategies are necessary together to obtain a good performance. We notice that it is much more difficult for audio models to visually predict the correct coordinates, having just rough horizontal information as input. Unlike the audio model, which does not have access to the visual modality, the audiovisual model finds a shortcut to localize the audiovisual spot, learning to rely more on the visual cue than the audio signal. We noticed that this occurs even though the network is trained to localize the audio source receiving as input the visual localization map of the speaker in the correct module, while the LED can be even 6° of visual angle far in position from the correct location.

#### Testing procedures

**Fig 3:**
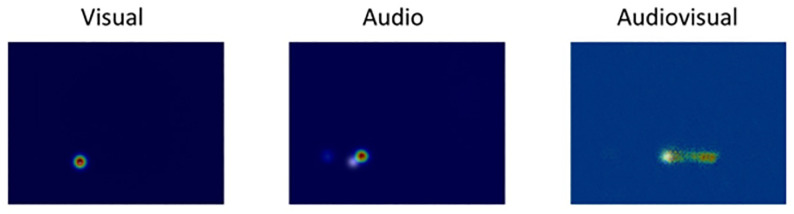
2D Localization maps obtained as output, respectively from visual, auditory, and audio-visual U-net. The corresponding stimulus is predicted to the basis of the corresponding input of the model.

To assess the validity of our testing procedures, we evaluated our models using qualitative and quantitative approaches. Qualitatively, we plotted the heat map with the stimuli predicted localization, and we overlapped it on the physical, correct position of the stimuli in the auditory, visual, and audiovisual localization tasks (Fig 3). The architecture was extremely precise when localizing visual stimuli horizontally and vertically centered on the correct coordinate. For audio localization, the signal was slightly misaligned on the vertical axis due to the missing vertical information; furthermore, auditory localization was not as accurate as visual localization, as it was not always correct on the horizontal for coarse spatial information contained in the beamforming features. Despite this, the auditory localization’s reduced accuracy and precision are similar to that of humans, as auditory localization is often more difficult than visual-spatial localization. For the audiovisual localization, its maximum is centered on the light position but creates a trace reaching the audio coordinate, clearly showing an attraction towards the visual stimulus and, consequently, a shift of the localization from the speaker to the LED light.

Quantitatively, Intersection over Union (IOU) is used to find the predicted module, corresponding to the higher IOU between the predicted location and ground-truth module position.

After finding the predicted module (from audio, visual or audiovisual models), we compute the shift between the correct and predicted module. This can be either zero if the prediction is correct or positive/negative if the prediction is wrong (towards the left/right of the ground-truth module, respectively). Then, we keep track of how many times we have such shifts and divide this number by the total number of trials to get the relative frequency of each possible shift between the predicted and the ground-truth module. This analysis was performed to evaluate the resulting predictions and assess how severe the wrong localizations are.

In the audiovisual test, we know the LED light shift compared to the audio position. Hence, we can figure out whether the prediction is pulled towards the LED’s light. In the test, the LED is always shifted to four modules to the speaker’s right or left. We divide the test data according to the right or left displacement and check the predicted module’s displacement. We notice that for the right displacement of the LED, prediction is pulled towards the speaker’s right, and similarly for left displacement. The peak of relative frequency distribution for right shift test data is centered at four modules on the right, and similar results are obtained for the left shift. These results show that even in our networks, we observe the same effect as that on human subjects: audio localization is pulled toward the visual stimulus.

## Results

### Human observers performance

**Fig 4:**
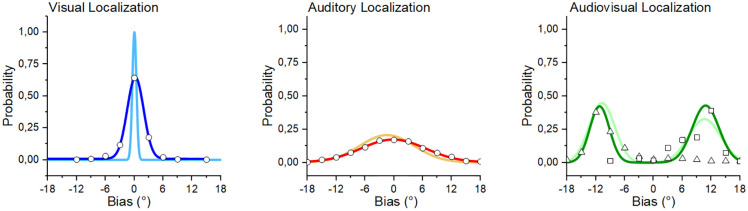
Gaussian fit for all three localization tasks. Darker colors represent the averaged human observer performance, while lighter colors represent the performance of the neural architecture. From the visual (A) and auditory (B) Gaussian fits, we extracted the X_a_, X_v_, σ_v_, and σ_a_ parameters that were successively used to model performance in the audio-visual (C) task. Scatter points indicate raw data for human participants (in Panel C, open triangles and open squares indicate performance in the visual-left and visual-right condition, respectively).

To evaluate performance in human observers in all tasks, we first calculated the perceived position of the stimulus as a function of its physical location (set at 0 by default). Then, we evaluated the difference between the two positions and the error response probabilities, averaging them across participants. Displacement distances were first calculated in modules and then converted into degrees of visual angle, considering the viewing distance of 180 cm. To define perceptual performance, for each probability, we fitted a Gaussian function through which we extracted two parameters: the center of the curve (X), which represented the most probable spatial estimation, and the standard deviation of the curve (σ), that represented perceptual variance (Fig 4).

We fitted Gaussian functions onto aggregate data obtained in humans or by using the architecture to assess localization performances. Then, we tested the probability that the fit was significantly better than the constant regression line y = k, i.e., we tested whether our data could have been approximated using a bell-shaped Gaussian distribution. All tests were performed considering an alpha level of 0.05. For visual localization, the fitted Gaussian function was significantly better than the function y = k (R^2^ = 0.98,) and returned as parameters X_v_ = 0.21° and σ_v_ = 1.7°, suggesting that human observers were extremely precise and accurate when localizing the position of the LED stimulus. For auditory localization, the fitted Gaussian function was also significantly better than the function y = k (R^2^ = 0.99), and it returned X_a_ = -0.15° and σ_a_ = 6.72°, indicating that human observers were less precise in estimating the position of the sound. Lastly, we evaluated performance in the audiovisual condition fitting two different Gaussian functions, one considering only the trials in which visual stimulations were shifted to the left of the sounds and one where they were shifted to the right of the sounds. In both conditions, Gaussian functions were significantly better than y = k (visual-left: R^2^ = 0.87; visual-right: R^2^ = 0.78). For the visual-left condition, we obtained X_avL_ = -11.235° and σ_avL_ = 2.04°, suggesting that stimulus localization was significantly biased towards the light position (as indicated by the negative value of X_avL_). Conversely, in the visual-right condition, we obtained X_avR_ = 11.34°, and σ_avR_ = 1.91°, suggesting a specular bias as highlighted by the positive value of the Gaussian peak.

### Neural architecture performance

To evaluate the Neural Architecture’s performance, we compared each stimulus’s reported spatial position as a function of its physical, actual position. We fitted a Gaussian function with every response probability similar to the average human observer. In all cases, fitted Gaussian functions were significantly better than the function y = k. After the fitting was achieved, we obtained the following parameters: for visual localization, X_v_ = 0°, σ_v_ = 0.52° (R^2^ = 1); for auditory localization: X_a_ = -1.44°, σ_a_ = 5.52° (R^2^ = 0.98); for audiovisual localization, in the visual-left condition: X_avL_ = -10.54° and σ_avL_ = 2.69° (R^2^ = 0.96), and in the visual-right condition: X_avR_ = 10.8° and σ_avR_ = 2.86° (R^2^ = 0.98).

Overall, the video model generalizes well on the test set because it learns from the accurate correspondence between input images and 2D Gaussian localization maps and easily localizes the LEDs’ position (Fig 4A).

The audio model spatial localization is not as precise as the video model localization because the beamforming performed using two microphones is coarse. For better localization, we would need to perform beamforming with more microphones located on a linear array or a planar array, which would have allowed a more accurate, yet more complex, 2D localization. Nevertheless, we presented stimuli changing their horizontal position while keeping their vertical coordinate constant, so a horizontal linear array was enough to assess the localization abilities of the model. Furthermore, we considered only two microphones to replicate human peripheral auditory input. The fact that auditory localization is not as effective as visual localization—but is significantly similar to the human’s—confirms our decision.

The drawback of the weak spatial information in the audio beamforming features is that our audio model is not generalizing well for the speaker localization task while in a different room acquisition configuration. This is caused by the fact that a little change in the audio wave’s reverberation (e.g., due to the room’s walls) dramatically impacts how the sound wave is recorded. Consequently, when there are some changes in the echoes, the beamforming features change a lot compared to those seen in the training.

For the audiovisual localization task, we obtained similar results to the ones measured in human participants: localization was strongly biased towards the position of the visual stimulus, suggesting that the model weighted the visual over the auditory input. Furthermore, the peaks of the two curves obtained fitting the Gaussian functions on the architecture probability outcome were placed around the peaks of the curves obtained considering the averaged human observer (Fig 4). This suggests that audiovisual integration for spatial localization followed similar patterns, at least at the empirical level.

### Model evaluation

**Fig 5:**
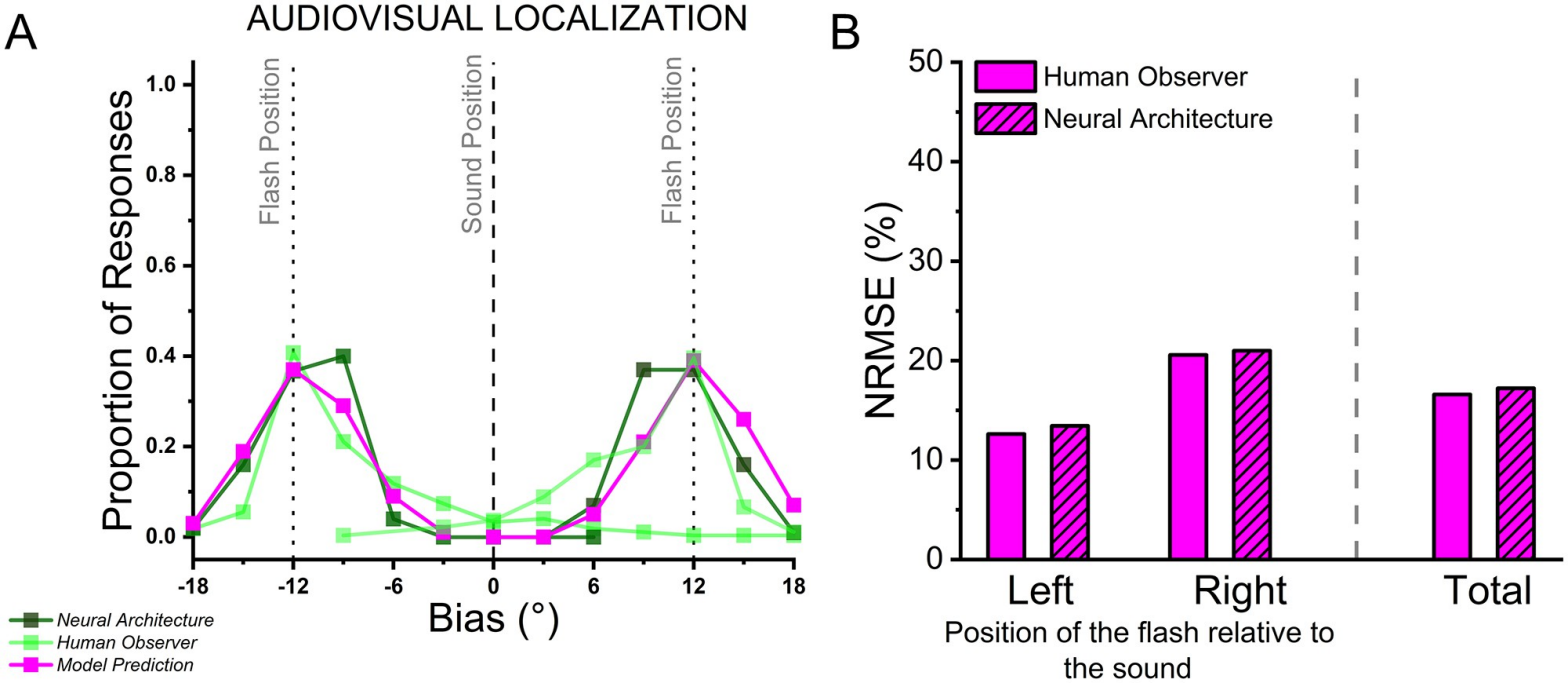
A) Performance of the optimal Bayesian observer (magenta line and points) compared with performance of the averaged human observer (light green) and the neural architecture (dark green). B) NRMSE values for the visual-left and visual-right conditions, and then reported considering the whole task.

To assess the validity of the architecture in the audiovisual localization task (i.e., in the task in which multisensory integration is supposed to occur), we compared the performance of both the human observers and the architecture against the predicted performance of the optimal Bayesian observer (see Methods above and Fig 5). To compare how well the Bayesian prediction could forecast empirical performance, we evaluated the Normalized-root-mean squared error (NRMSE). The NRMSE is the normalized version of the root-mean-square error (RMSE), which measures the difference between a model’s predicted values and the empirically observed one. We normalized the RMSE using the range of the observed values (Eq 3) and expressed it as a percentage.

Our analysis highlighted two notable findings. First, NRMSE values were similar for the averaged human observer and the neural architecture, both when separately considering audiovisual displacements (visual-left: NRMSE_human_ = 12.62%, NRMSE_architecture_ = 13.43%; visual-right: NRMSE_human_ = 20.57%, NRMSE_architecture_ = 20.99%) and when considering averaged performance (NRMSE_human_ = 16.6% and NRSME_architecture_ = 17.21%, respectively), suggesting that the architecture’s behavior is an accurate approximation of audiovisual integration processing for spatial localization in humans. Second, the average human observer and the architecture NRSME were fairly low, accounting for approximately 17% of the residual variance.

To further support our evaluation, we directly compared the raw performance obtained from the neural architecture and human participants. To do so, we compared response probabilities across the two audiovisual conditions, testing whether human observers and the architecture provided different response patterns. Thereby, we performed non-parametric statistical analyses considering as dependent variable the proportion of correct responses (shown in Fig 5, dark and light green scatter points: for this analysis, we considered only the points in which the two curves overlap), and as a main factor the observer (either humans or the architecture). Overall, our analysis highlighted that humans and the architecture performed similarly, both in the left (Mann-Whitney U = 25, n_Architecture_ = 8, n_Humans_ = 8, *p* = 0.49, r_rb_ = -0.22) and right (Mann_Whitney U = 19, n_Architecture_ = 7, n_Humans_ = 7, p = 0.52, r_rb_ = -0.24) audiovisual conditions.

Considering these results, we concluded that—from an empirical standpoint—our architecture significantly behaved as the averaged human participants, i.e., as the optimal Bayesian observer.

## Discussion

With the current work, we developed a neural architecture to mimic humans’ ability to build and create audiovisual spatial representations for source localization. Our architecture empirically replicated the observed perceptual performance in humans, overcoming two significant computational issues related to multisensory integration: the recoding and the statistical inference problems.

The recoding problem emerges considering that the brain must convert different sensory signals into a common metric before successful integration. Since sensory information coming from different sensory modalities is originally encoded within different frames of reference [1–7], they must be first rescaled to be comparable. For audiovisual localization, auditory and visual information is considered on a more abstract level, i.e., spatially, rather than on an overly specific, less generalizable level, i.e., through a retinotopic-tonotopic coordinate system. This conceptualization aligns with evidence supporting an occipital cortical network debuted on spatial localization [32]. Despite the critical role of vision in the development of spatial representations, supported by its disruption in early and congenitally blind individuals [33], cortical activation of occipital areas—that are inherently visual—is observed when localizing the spatial position of a sound [34]. Such cortical activation favorably witnesses the existence of recoding mechanisms, as it is necessarily evoked due to the spatial position of the auditory stimulus rather than its tonotopic features [35, 36].

Our neural architecture only combines auditory and visual information after converting it to an ordinary symbolic level to create audiovisual spatial representation. For this reason, it is necessary to process the inputs from the two different sensory modalities to achieve a more abstract level before integrating them within the same percept. The abstract representation is a spatial feature map extracted from the pre-trained video model, specialized on our data for the visual modality. For the audio model, it is the spatial map reconstructed by the decoder of the U-Net model, which also has an abstract meaning as it is reconstructed after an embedding representation. Then, the visual feature map can be fused through concatenation to the aural map after bringing both to a similar abstract level. Even though the conversion of both signals is inevitable before their comparison through the work of the architecture, it is undeniable that it is not possible to reach the most abstract semantic representation achievable, as we keep a map to preserve the spatial meaning of the inputs. Nonetheless, after the combination, both signals are processed by convolutional layers, which allow the refinement of their fusion and the combined spatial localization.

Similarly, the brain intertwines inputs from different sensory modalities at the cortical level [36, 37], even though multisensory integration partially occurs at the sub-cortical level [37]. In primary cortices, sensory information—initially encoded through different frames of reference—is combined before being used for decision-making processes [38]. In support of this view, many studies [34–36, 39, 40] highlighted cortical activation patterns that can be considered both domain-dependent (e.g., related to space) and modality-independent (e.g., unrelated to the involved sensory channels).

For spatial perception, cortical occipital activation was also found when participants focused on the spatial position of various stimuli. In line with what was suggested a few lines above, cortical occipital activation is found when participants are required to localize the spatial position of sounds [32, 34]. The activation of occipital areas in response to sounds, i.e., unrelated to the sensory modality involved, is a significant indicator that spatial perception is not tied to single sensory inputs. Instead, spatial perception is combined and refined through an occipital network [40], which combines audiovisual information to localize stimuli across the environment.

To achieve optimal integration, humans must also determine statistical probabilities for each sensory cue, depending on the reliability of the information itself at a given moment. This results to be the statistical inference problem, and to date, it is still unclear how the brain physiologically manages to solve it. In a notorious work, Ernst and Banks [8] proposed that sensory information is integrated into a statistically optimal fashion to minimize the perceptual variance of the final estimation. Consequently, multisensory integration has been modeled using Maximum-likelihood estimations (MLE), through which Bayesian probabilities are applied to each sensory cue [41]. When auditory and visual information is available to build spatial representations of the environment, the brain strongly relies on visual rather than auditory cues [11].

For this reason, in an ambiguous situation (e.g., when attending a ventriloquism show), source localization is strongly driven by the visual information that ‘attracts’ our percept (and therefore, we experience the voice coming out from the puppet rather than the ventriloquist). Notably, when testing the architecture, we obtained a performance similar to the one found with humans in both unimodal and bimodal localization tasks. First, the architecture was more precise in the visual localization task than in the auditory localization task (Fig 4A and 4B): this was mandatory to reproduce human-like audiovisual integration since visual information drives source localization. Second, audiovisual localization for the architecture was strongly biased toward the position of the visual stimulus (Figs 4C and 5), in line with what was expected in humans and the mathematical model prediction (see *Model Evaluation*).

Even though our model could theoretically tackle both the recoding and the statistical inference problems, it is hard to bridge it to precise neurophysiology. The most challenging obstacle in providing a reliable physiological interpretation is linked to the model configuration itself. Although inspecting hidden layers might be considered a possible solution, it does not provide any mechanistic insight. Specifically, the interpretability of neural networks is an open issue in the Machine Learning community [42], and, as a general rule, the more extensive the network, the lower its intelligibility. Combining neurons with non-linear activations over several hidden layers is essentially a way to approximate complex data relationships that cannot be written with a mathematical formula. Even though gradient-based methods [43] can provide some insights into the spatial arrangement of low-level or semantic features extracted by neural networks, the simple localized stimulus (as in our case) would only produce very naïve local stimuli within the architecture.

In light of the inability to create a physiological bridge between the architecture and the human observer, we recognize this limit, and we argue that it is not the main objective of this work. Ultimately, we aimed to provide a tool to model behavioral data rather than dip into physiological inferences. Nevertheless, we also argue that, despite its limits, the strengths of this work are multiple.

First, we release both the model’s code and the dataset used for source spatial localization and the ventriloquist effect study. We foster the value of this dataset in conjunction with the model, as stimuli included in the dataset are a former replica of stimuli often used in psychophysics (e.g., [44, 45]). All stimuli included in the dataset were manually recorded within a controlled experimental setting, through which reverberation and luminosity were strictly monitored. Furthermore, within the dataset, we had stimuli that are simpler than those typically used to train audiovisual localization models (e.g., [46]), which often rely on ecological and semantic validity. Conversely, given their simplicity, stimuli included within our dataset are usually more suitable to investigate basic perceptual mechanisms. Thanks to this peculiarity, the dataset we share represents a more direct computational comparison with human performance. Second, directly modifying the dataset’s items (e.g., blurring the image or muffling the sound) will potentially change how the architecture integrates auditory and visual information, allowing performance modeling in specific populations (e.g., visually or auditory-impaired people). Potentially, this highly customizable approach can be used to predict spatial localization performances just tailoring, with extreme accuracy, the sensory impairment of a given individual. For example, using both our model and the dataset, one can replicate the performance in a patient with a scotoma by using their campimetry, recreating the visual blindspots with extreme accuracy. For these reasons, our architecture will be a valuable tool to better understand multisensory integration mechanisms and properly model source localization performance in individuals with and without sensory impairments.
